# Abdominal Cerebrospinal Fluid Pseudocyst as a Complication of Ventriculoperitoneal Shunt Placement

**DOI:** 10.7759/cureus.9363

**Published:** 2020-07-23

**Authors:** Ted George O Achufusi, Philip Chebaya, Sekou Rawlins

**Affiliations:** 1 Internal Medicine, State University of New York Upstate Medical University, Syracuse, USA; 2 Gastroenterology, State University of New York Upstate Medical University, Syracuse, USA

**Keywords:** abdominal pseudocyst, ventriculoperitoneal shunt, hydrocephalus, ascites

## Abstract

The abdominal cavity has long been used for absorption of cerebrospinal fluid (CSF) in patients with hydrocephalus. Although the procedure is quite common, there are complications that can potentially arise following ventriculoperitoneal (VP) shunt insertion. Here, we report a case of a 39-year-old female patient in which a large abdominal pseudocyst was developed as a complication of VP shunt placement. Ultrasonographical evaluation of the abdomen showed a well-defined cystic mass lesion later confirmed on CT abdomen. She subsequently underwent surgical excision of the pseudocyst with resolution of previous symptoms. Clinicians should be aware of this complication since early diagnosis improves outcome and reduce patient's suffering and distress.

## Introduction

Ventriculoperitoneal (VP) shunt placement is a common neurosurgical procedure performed for the management of hydrocephalus. Although rare, there are potential complications that might arise from the procedure, including abdominal wall perforation, peritonitis, and ascites [1]. Abdominal pseudocyst formation is an uncommon but well-described complication of the procedure. Herein, we present the case of a young female patient who presented to our hospital with abdominal distension and pain and was subsequently found to have a massive cerebrospinal fluid (CSF) pseudocyst attributed to VP shunt malfunction.

## Case presentation

**Figure 1:**
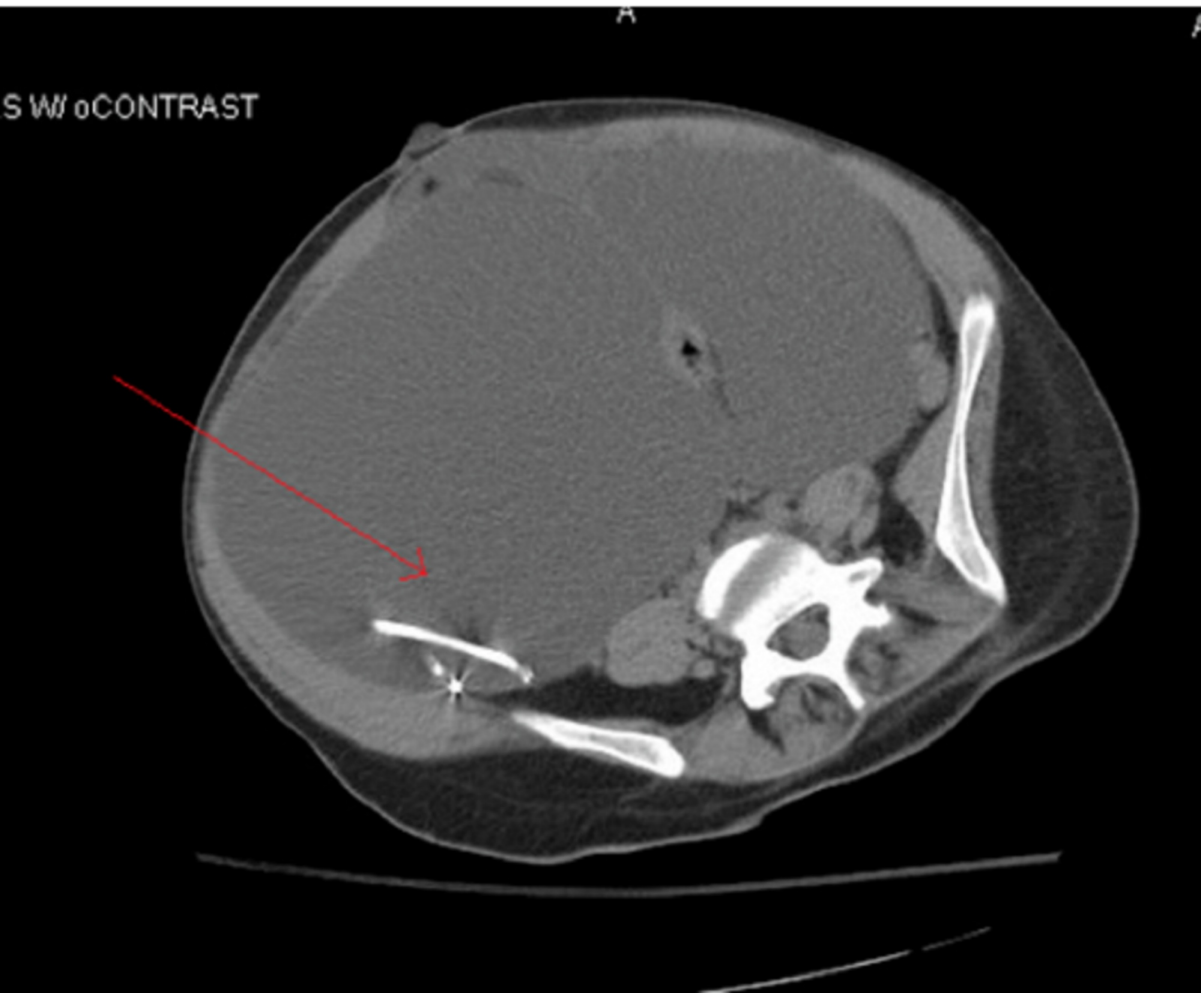
Axial CT showing abdominal fluid collection adjacent to ventriculoperitoneal shunt catheter tip

A 39-year-old Caucasian female with a history of cerebral palsy with spastic quadriplegia and congenital hydrocephalus with VP shunt presented to our institution for rapidly developing abdominal distension, pain, and coffee-ground emesis. On examination, the patient was lethargic and in obvious discomfort. Her abdomen was markedly distended with rebound tenderness and decreased bowel sounds. Abdominal ultrasound revealed severe free fluid in the abdomen which was confirmed on CT of the abdomen. Upper endoscopy was negative for active bleeding, varices, or ulcers. Diagnostic paracentesis showed serum-ascites albumin gradient (SAAG) > 1.1, total protein (TP) < 2, and clear yellow fluid. There was no evidence of cirrhosis on imaging with patent hepatic vein, splenic vein, and inferior vena cava (IVC). She had an echocardiogram that did not show any cardiomyopathy or restrictive pericarditis. Fibrosure showed F0, no hepatic fibrosis. Repeat CT later on in that admission showed large amount of intra-abdominal partially loculated fluid suspicious of CSFoma (Figure 1).

Neurosurgery was consulted and did not feel that CSFoma was likely. She followed up with GI outpatient for workup of the ascites with all workup for hepatic or portal vein etiology being negative, including a transjugular liver biopsy. It was determined that the fluid collection was not hepatic in nature and that CSFoma needed to be further investigated. She continued to experience worsening distension and foot swelling and again presented to the ED one week following discharge. She was seen by neurosurgery who requested additional abdominal imaging. Upon review of imaging studies, including CT of the abdomen/pelvis (A/P), it seemed CSFoma was caused by loculations and septations around the peritoneal end of the VP catheter. She was taken to the operating room for attempted laparoscopic drainage of her intraperitoneal CSF cysts. That was followed by open conversion to repair a loop of bowel that was inadvertently dissected during the laparoscopic procedure. There were multiple adhesions seen during the procedure with several anastomoses created. At the same time, one nonfunctioning peritoneal shunt was removed, and a second was replaced in the right upper quadrant. She recovered very well from her surgery and there have been no additional complications following the procedure.

## Discussion

VP shunt placement is a procedure performed to drain excessive CSF which otherwise can lead to serious complications, including increased intracranial pressure and hydrocephalus formation. Pseudocyst formation due to shunt placement is mostly seen in the abdomen as most ventricular drains are placed in the peritoneal cavity. Inadequate absorption, blockages, and abdominal adhesions are some of the common complications leading to accumulation of CSF at the end of the catheter with subsequent pseudocyst formation [2].

Signs of VP shunt malfunction include headache, nausea, altered level of consciousness, and other findings commonly observed in those with elevated intracranial pressures. Abdominal complications related to VP shunt placement include pseudocysts, intraabdominal infections (e.g. abscess), and bowel perforation [1]. Pseudocysts are more common than ascites, and present as a localized abdominal mass. Those with pseudocysts present with abdominal discomfort and diffuse abdominal tenderness. Depending on the size of the pseudocyst, symptoms of bowel obstruction may arise, including nausea and vomiting. CT scan of the abdomen remains the most common diagnostic modality, although ultrasound has also been used in some cases. CT scan is often more useful in distinguishing those presenting with severe abdominal pain as it can help identify other etiologies, such as appendicitis, diverticulitis, abdominal abscess, or bowel obstruction. Pseudocysts can be identified on CT abdomen as large fluid-filled collections delimited by a thin wall adjacent to the catheter tip (Figure 1). Differential diagnoses of an abdominal cystic mass include pancreatic pseudocyst, lymphocele, cystic lymphangioma, mesenteric cyst, benign cystic teratoma, seroma, cystic spindle-cell tumor, and cystic mesothelioma [3]. In this case, the absence of any infectious symptoms or history of pancreatic disease combined with history of VP shunt placement significantly narrowed the differential diagnoses.

The exact pathogenesis of abdominal pseudocyst remains unknown; however, three different mechanisms have been proposed: chronic infection, foreign body reaction, and particle like protein in CSF [4]. The time for an abdominal pseudocyst to develop from the last shunting procedure ranges anywhere from three weeks to five years, with the longest reported cases in literature being eight years and fifteen years [5,6]. The question of optimal treatment of abdominal pseudocysts has not been fully answered. Treatment largely depends on etiology, patient presentation, and clinical manifestations. Currently, several treatment methods have been described, including CT-guided fluid aspiration, paracentesis, and laparotomy with excision of cystic walls. In this case, our patient underwent open laparotomy cyst removal with reposition of the distal shunt catheter.

## Conclusions

Abdominal pseudocyst remains a rare but important complication of VP shunt placement and is likely attributed to a low-grade inflammatory process. Clinicians other than neurosurgeons and pediatricians should be aware of this complication and keep it on the differential in those with history of VP shunt placement presenting with acute abdomen. Early recognition and intervention have been shown to improve clinical outcomes and limit unnecessary and costly diagnostic workup.

## References

[1] Kashyap S, Ghanchi H, Minasian T, Dong F, Miulli D (2017). Abdominal pseudocyst as a complication of ventriculoperitoneal shunt placement: review of the literature and a proposed algorithm for treatment using 4 illustrative cases. Surg Neurol Int.

[2] Habibi S, Jenkins C, Natt B (2015). Medical image of the week: 'CSFoma'. Southwest J Pulm Crit Care.

[3] Pernas JC, Catala J (2004). Case 72: pseudocyst around ventriculoperitoneal shunt. Radiology.

[4] Mobley LW 3rd, Doran SE, Hellbusch LC (2005). Abdominal pseudocyst: predisposing factors and treatment algorithm. Pediatr Neurosurg.

[5] Gmeiner M, Wagner H, Van Ouwerkerk WJR, Senker W, Holl K, Gruber A (2018). Abdominal pseudocysts and peritoneal catheter revisions: surgical long-term results in pediatric hydrocephalus. World Neurosurg.

[6] Comba A, Gülenç N, Çaltepe G, Dağçınar A, Yüce O, Kalaycı AG, Ulus A (2013). Ascites and abdominal pseudocyst: two uncommon ventriculoperitoneal shunt complications in two cases. Turk J Pediatr.

